# Revolutionizing Donor Heart Procurement: Innovations and Future Directions for Enhanced Transplantation Outcomes

**DOI:** 10.3390/jcdd11080235

**Published:** 2024-07-27

**Authors:** Marc Leon

**Affiliations:** Department of Cardiothoracic Surgery, Stanford University School of Medicine, 300 Pasteur Drive, Falk CVRB, Stanford, CA 94305, USA; lianghl@stanford.edu

**Keywords:** heart transplantation, donor heart procurement, donation after circulatory death, extended-criteria donors, artificial intelligence

## Abstract

Heart failure persists as a critical public health challenge, with heart transplantation esteemed as the optimal treatment for patients with end-stage heart failure. However, the limited availability of donor hearts presents a major obstacle to meeting patient needs. In recent years, the most groundbreaking progress in heart transplantation has been in donor heart procurement, significantly expanding the donor pool and enhancing clinical outcomes. This review comprehensively examines these advancements, including the resurgence of heart donation after circulatory death and innovative recovery and evaluation technologies such as normothermic machine perfusion and thoraco-abdominal normothermic regional perfusion. Additionally, novel preservation methods, including controlled hypothermic preservation and hypothermic oxygenated perfusion, are evaluated. The review also explores the use of extended-criteria donors, post-cardiopulmonary resuscitation donors, and high-risk donors, all contributing to increased donor availability without compromising outcomes. Future directions, such as xenotransplantation, biomarkers, and artificial intelligence in donor heart evaluation and procurement, are discussed. These innovations promise to address current limitations and optimize donor heart utilization, ultimately enhancing transplantation success. By identifying recent advancements and proposing future research directions, this review aims to provide insights into advancing heart transplantation and improving patient outcomes.

## 1. Introduction

Heart failure is a severe health threat, affecting a vast population. Globally, there are at least 56.2 million individuals living with heart failure. However, this figure is likely significantly underestimated, particularly in low-resource regions, due to data gaps [1]. In 1967, Dr. Christiaan Barnard performed the world’s first human heart transplant in South Africa [2]. Shortly thereafter, in 1968, Dr. Norman Shumway completed the first heart transplant in the United States at Stanford University Hospital [3]. Over the past fifty years, heart transplantation has become recognized as the optimal treatment for end-stage heart failure. However, the number of donor hearts available for transplantation remains far below the demand from end-stage heart failure patients. In recent years, significant technological advancements in heart transplantation, particularly in donor heart procurement techniques, have been notable, substantially expanding the donor heart pool and enabling more patients to undergo heart transplantation. This review aims to summarize the current status and advancements in donor heart procurement techniques in the United States and to advocate for future directions in this field.

## 2. Recent Trends and Outcomes in U.S. Heart Transplantation

According to public data from the United Network for Organ Sharing (UNOS) (https://unos.org/data/, accessed on 2 July 2024), the number of heart transplants in the United States was 3818 in 2021, 4111 in 2022, and 4545 in 2023. The primary causes for transplantation were cardiomyopathy, coronary artery disease, and congenital heart disease. From 2011 to 2022, the mortality rate post-heart-transplantation in U.S. adults remained stable or slightly improved, except for the 10-year mortality rate, which increased from 35.7% in 2011 to 37.4% in 2022 [4]. The 6-month post-transplant mortality rate is 7.3%, the 1-year rate is 9.2%, the 3-year rate is 15.3%, and the 5-year rate is 19.9%. Among adult heart transplant recipients between 2015 and 2017, those with congenital heart disease had the lowest short-term and long-term survival rates, with a 3-month survival of 89.7% and a 5-year survival of 75.0% [4]. Recipients with cardiomyopathy had the best 1-year survival rate at 92.4% and a 5-year survival rate of 82.2% [4]. In 2022, the majority of adult heart transplant recipients were aged 50 to 64 (46.6%). Since 2011, the number of recipients aged 65 and older and those aged 18 to 34 has increased by 122.1% and 104.9%, respectively [4]. Notably, the number of heart transplants in recipients aged 65 and older has shown a yearly increase, with 730 cases in 2021, 741 in 2022, and 841 in 2023. UNOS data show that the 1-year survival rate for recipients aged 65 and older is 88.4% (95% CI: 86.7–89.8), and the 5-year survival rate is 75.2% (95% CI: 72.7–77.6), indicating satisfactory outcomes. Studies have shown that the 5-year survival rate post-heart-transplantation for recipients aged 70 and older is comparable to that of younger patients [5]. However, older recipients are more prone to stroke and are more likely to die from infections or malignancies, while the likelihood of dying from acute rejection is lower compared to younger recipients [5,6].

In 2018, UNOS implemented significant reforms to the donor allocation system, prioritizing heart allocation to candidates supported by temporary mechanical circulatory support (MCS) over those with long-term MCS, reflecting the more urgent medical needs of the former [7]. This reform significantly reduced the median wait time for candidates on extracorporeal membrane oxygenation (ECMO) from 10 days to 5 days and improved the 30-day and 6-month survival rates from 76.4% to 94.2% and from 74.6% to 90.6%, respectively. Concurrently, the proportion of heart transplant recipients using long-term left ventricular assist devices (LVADs) decreased from 41.8% to 21.2%, and the proportion of candidates using LVADs while on the waitlist also decreased from 14.5% to 10.8%. Moreover, for critically ill status 1 and 2 candidates, the donor heart allocation radius was expanded to 500 miles (approximately 805 km) to more equitably utilize heart transplantation resources. The system reform significantly impacted wait times, heart procurement distances, and ischemic times. The average wait time decreased from 93 days to 41 days; the median heart procurement distance increased from 83 miles (approximately 134 km) to 225 miles (approximately 362 km); and the median ischemic time extended from 3 h to 3.5 h. Despite the increased hospitalization duration, the incidence of acute rejection before discharge remained stable (11.9% vs. 11.7%). Postoperative 30-day, 90-day, and 180-day survival rates remained consistent before and after the policy change, alleviating concerns about potentially worse outcomes for high-risk patients. 

## 3. Application of Donation after Circulatory Death (DCD) Hearts

The most significant advancement in heart transplantation in recent years is the surge in the use of DCD hearts. Organ donation can occur after a patient’s death, defined as either (1) irreversible cessation of brain function or (2) permanent cessation of circulatory function [8]. Donation after brain death (DBD) involves donors confirmed through repeated neurological examinations to have lost brainstem reflexes and cerebral blood flow. DCD donors, however, are those with irreversible severe neurological damage who do not meet the criteria for brain death; their circulatory cessation is confirmed through physical examination or intra-arterial pressure monitoring [8]. The first heart transplant in 1967 used a DCD donor heart [2]. However, concerns about warm ischemic injury led to a pause in DCD heart use, and adult heart transplants relied solely on DBD donors for decades. To address the gap between transplant demand and available hearts, Australian surgeons resumed DCD heart use in 2014 [9]. In the United States, clinical trial results published in 2023 showed that early survival rates for DCD heart transplants were comparable to those for DBD heart transplants [10]. The trial, involving 180 patients randomly assigned in a 3:1 ratio to receive DCD or DBD heart transplants, showed a 6-month survival rate of 94% for the DCD group versus 90% for the DBD group. The incidence of severe primary graft dysfunction (PGD) was 15% in the DCD group compared to 5% in the DBD group. Other studies confirmed similar 6-month survival and complication rates between DCD and DBD hearts, ranging from 91% to 95% [11,12,13,14]. The use of DCD donors could significantly increase heart transplant numbers and reduced patient wait times [15]. Shivank Madan and colleagues estimated that DCD donors could add 300 adult heart transplants annually in the United States [14]. From 2018 to 2023, the use of DCD hearts in the United States rose rapidly, from 0 cases in 2018 to 612 in 2023. This increase is driven by advancements in donor heart reanimation, evaluation, and preservation techniques. Unlike DBD hearts, DCD hearts undergo warm ischemia and are non-beating at procurement. Reanimation and evaluation of DCD hearts rely on two strategies: direct procurement using machine perfusion (DP-MP) or thoraco-abdominal normothermic regional perfusion (TA-NRP) followed by heart procurement. Post-procurement preservation includes normothermic machine perfusion (NMP), controlled hypothermic preservation using the SherpaPak Cardiac Transport System (SCTS), hypothermic oxygenated perfusion (HOPE), and traditional static cold storage (SCS). 

The DCD heart donation process typically follows the controlled withdrawal of life-sustaining therapy (WLST) [8]. This decision must be made independently of the potential for organ donation and by professionals not associated with the transplant team or potential recipients. Throughout WLST, comprehensive care ensures patient comfort. The first declaration of death occurs when circulation ceases. If organ donation proceeds, a 5-min observation period ensures no spontaneous return of circulation [16]. Following this period, a second declaration of death allows the transplant team to begin organ procurement [17]. The observation window ranges from 60 to 120 min; if death is not declared within this time, organ donation will not proceed, and patient care continues. Warm ischemic time (WIT) is the most critical factor affecting donor heart quality. Standards for WIT and permissible time windows vary among institutions [18]. For instance, Stanford University Hospital considers functional WIT to start when systolic blood pressure drops to 50 mmHg after WLST and end with cold cardioplegia perfusion [19]. The functional WIT window is limited to 30 min. If the donor is not declared dead within this time, the heart is not viable. If systolic pressure rises above 50 mmHg, functional WIT timing resets. Most WIT occurs before death declaration and is beyond the transplant team’s control. However, the interval between death declaration and heart perfusion initiation can be minimized through skilled surgical techniques and team coordination. Thus, DCD heart procurement should be conducted by experienced surgical teams.

## 4. Ex Vivo Normothermic Perfusion of Donor Hearts—TransMedics Organ Care System (OCS)

The TransMedics Organ Care System (TransMedics, Inc., Andover, MA, USA) is the sole ex vivo heart perfusion platform approved by the U.S. Food and Drug Administration (FDA) for clinical use [20]. This portable extracorporeal system is designed to resuscitate, preserve, and assess donor hearts in a near-physiologic, normothermic, and beating state. It supports direct procurement of DCD hearts using DP-MP, making it the most widely adopted technology for DCD hearts. Additionally, the TransMedics OCS extends the ex vivo safety time window for both DBD and DCD hearts, enabling long-distance transportation.

**Figure 1:**
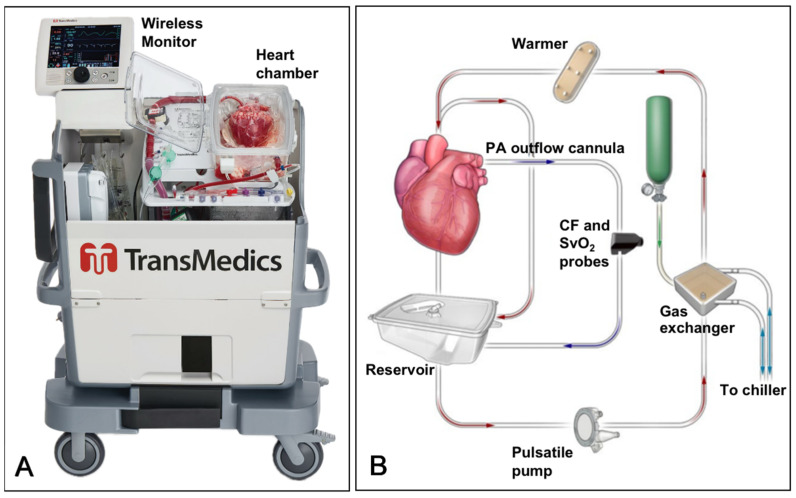
TransMedics OCS Heart System. (**A**). TransMedics OCS device, front view with the cover removed. (**B**). Schematic diagram of the OCS working principle. The OCS circulates warmed, oxygenated perfusate through the heart preservation module circuit. Blood, supplemented with TransMedics solutions, is pumped from the reservoir through an oxygenator and warmer. This warm, oxygenated blood is directed to the aorta to perfuse the coronary arteries. Deoxygenated blood returns from the coronary circulation to the right atrium, passes through the tricuspid valve to the right ventricle, and is ejected through the pulmonary artery back to the reservoir for recirculation. CF: coronary flow; PA: pulmonary artery; SvO_2_: mixed venous oxygen saturation. The images are sourced from publicly available materials and are used with permission from TransMedics, Inc.

The OCS Heart System (Figure 1A) includes the console with a perfusion pump, perfusion set, and solution set for preservation. For both DBD and DCD hearts, 1.2–1.5 L of donor blood is collected and the heart is perfused with cold cardioplegic solution before excision. Afterward, the heart is connected to the OCS aortic port, with the pulmonary artery cannula returning blood to the reservoir. Oxygenated blood at 34 °C is pumped into the aorta to achieve coronary perfusion. Blood returns via the coronary sinus, right atrium, right ventricle, and pulmonary artery to the reservoir, where it is re-oxygenated and recirculated through the aorta, establishing a blood circuit (Figure 1B). Insulin, antibiotics, albumin, and other agents are added to the perfusion circuit. The heart typically resumes beating shortly after normothermic perfusion begins. Hemodynamic parameters, such as aortic pressure and coronary flow, are continuously monitored. Electrolyte imbalances and acid–base disturbances are corrected as needed. The assessment of the heart’s suitability for transplantation includes satisfactory contractility, stable hemodynamics, decreasing lactate levels, and higher arterial than venous lactate, indicating good myocardial lactate uptake and adequate myocardial perfusion [10,21]. When the donor heart is transported to the recipient’s operating room, it is first cooled, the pulmonary artery cannula is removed, and the inferior vena cava is opened. The OCS blood pump is then turned off, and cold cardioplegia is administered via the aortic port to induce cardiac arrest. The donor heart is then removed from the OCS for transplantation.

The use of the TransMedics OCS has significantly advanced DCD heart transplantation by enabling optimal maintenance and assessment of the heart post-circulatory-cessation [22]. Additionally, for both DBD and DCD donor hearts, this technology offers several key advantages: reducing cold ischemic time, evaluating extended-criteria donor (ECD) hearts, extending safe ex vivo time, and expanding the donor pool. Studies on the TransMedics OCS show that its short-term and long-term survival outcomes are comparable to those of traditional SCS techniques. A meta-analysis, which included 12 heart transplant studies with a total of 741 donor hearts (260 using the OCS), demonstrated the safety and efficacy of the OCS in both DBD and DCD donations, especially for long-distance transport [23]. The early and late survival outcomes were similar between the OCS and SCS groups. Notably, the 30-day survival rates were 97.4% for the OCS–DBD group, 96.6% for the OCS–DCD group, and 95.7% for the SCS–DBD group, with no significant differences in short-term survival, hospital stays, PGD, or rejection episodes. The pooled 1-year survival rates were 84.2% for the OCS–DBD group, 89.3% for the OCS–DCD group, and 87.0% for the SCS–DBD group. The pooled 4-year survival rates were 82.2%, 85.3%, and 80.3%, respectively. The OCS EXPAND trial was the first prospective, single-arm, multi-center pivotal study in the United States to evaluate clinical outcomes using the OCS for extended-criteria DBD donor hearts [24]. Inclusion criteria for donor hearts included an anticipated total ischemic time (from donor aortic cross-clamp to reperfusion in the recipient) of over 4 h, or over 2 h with additional risk factors such as donor age ≥ 55 years, donors aged 45–55 without coronary angiography, a period of donor unconsciousness or circulatory arrest ≥ 20 min with stable hemodynamics at heart assessment, left ventricular (LV) septal or posterior wall thickness of 13–16 mm, or LV ejection fraction (LVEF) of 40–50%. Of the 173 DBD donor hearts perfused with the OCS, 150 (87%) were successfully transplanted, while 23 (13%) did not meet transplant criteria. The 30-day survival rate for recipients was 97%, with a severe PGD incidence of 6.7%. The average number of serious adverse events related to the donor heart within 30 days was 0.17 (95% CI: 0.11–0.23). Recipient survival rates at 6 months, 12 months, and 24 months were 93%, 89%, and 86%, respectively [24]. In conclusion, the OCS technology has the potential to significantly increase the utilization of DBD donor hearts and the overall number of heart transplants.

Despite the rapid increase in the use of TransMedics OCS for heart transplantation in the United States, several challenges remain. The high cost of the device, along with the need for a larger specialized organ procurement team, extensive personnel, equipment, and technical support, are noteworthy concerns [20,25,26]. Furthermore, prolonged ex vivo heart perfusion may lead to adverse effects such as myocardial edema. When the heart beats under OCS perfusion, it lacks preload, which is not physiological, making contractility assessment during this period potentially inaccurate for predicting post-transplant function. Additionally, the use of lactate as a biochemical marker for predicting donor heart quality has been questioned. Ongoing research aims to optimize perfusion solutions and develop more sensitive and effective heart assessment techniques [27].

## 5. Thoraco-Abdominal Normothermic Regional Perfusion

The TA-NRP is a technique used for recovering DCD donor hearts and assessing their viability. Unlike the DP-MP technique represented by the TransMedics OCS, TA-NRP allows for the in situ recovery of the heart and its evaluation under physiological conditions, serving as an effective alternative to DP-MP.

The surgical procedure for TA-NRP varies slightly among medical institutions, but the basic steps remain consistent [28]. After the DCD donor is declared dead for the second time, the surgical team quickly performs a thoracotomy and opens the pericardium. A right atrial cannula is inserted to drain venous blood and empty the heart. All aortic arch vessels are clamped to prevent cerebral perfusion. The ascending aorta is cannulated and connected to veno-arterial ECMO (VA-ECMO) to establish extracorporeal circulation, restoring normothermic oxygenated blood perfusion to the body except for the brain. To further drain cerebral blood, a cannula can be inserted into the distal end of the innominate artery. Additionally, cannulation of the main pulmonary artery can be performed to reduce left heart load. After establishing VA-ECMO, the donor is intubated and connected to a ventilator to restore pulmonary ventilation. The initial target flow rate for TA-NRP is typically set at 5 L/min. If ventricular fibrillation occurs, in situ defibrillation is performed. Under VA-ECMO support, the heart usually regains sinus rhythm shortly after perfusion is restored, electrolyte imbalances and acid–base disturbances can be corrected, and vasopressors may be administered to maintain a mean arterial pressure above 60 mmHg. Based on the recovery of heart function, TA-NRP generally lasts for 30 to 60 min. After ECMO weaning, the heart’s function is assessed. Some institutions use Swan–Ganz catheters and transesophageal echocardiography for this evaluation, while others rely on the surgeon’s visual and tactile assessment. If the donor’s heart is deemed suitable for transplantation, cold cardioplegia is administered to arrest the heart, and the subsequent procurement process follows the same protocol as for DBD hearts. After excision, the heart can be preserved using traditional SCS techniques, though an increasing number of physicians are opting for the SCTS.

Compared to DP-MP techniques, TA-NRP significantly reduces ischemic time between donor circulatory cessation and heart resuscitation, allows functional assessment of the heart under physiological conditions, and is more cost-effective [18,29]. Marian Urban and colleagues at the University of Nebraska Medical Center found that the direct cost per heart using TA-NRP was $155,955, compared to $223,399 for DP-MP [26]. Despite the lack of statistical significance in cost difference, TA-NRP showed better contribution margin, contributing $242,657 compared to $175,768 for DP-MP. The clinical efficacy of TA-NRP is well recognized. Vanderbilt University Medical Center reported on 15 DCD donor hearts procured using TA-NRP, preserved with SCS [30]. The average TA-NRP duration was 56 min with an average ischemic time of 183 min. All recipients survived 30 days post-transplant, with six experiencing mild PGD and three moderate PGD, and no severe PGD. Postoperative echocardiography showed all donor hearts had an LVEF exceeding 55%. New York University Langone Health reported eight DCD heart transplants using TA-NRP, including six heart transplants, one heart–lung transplant, and one heart–kidney transplant [13]. The heart–lung transplant recipient required VA-ECMO support due to initial lung dysfunction but was successfully weaned off by the third postoperative day. The median follow-up period was 304 days, with a 100% recipient survival rate. A retrospective study in the United Kingdom compared 57 DCD donor hearts procured using TransMedics OCS and 19 using TA-NRP [31]. There were no statistical differences in 30-day, 90-day, and 1-year survival rates, heart function, inotropic support, or MCS use between the groups. Additionally, no significant differences were found in survival rates between DCD hearts procured using either TA-NRP or TransMedics OCS and traditional DBD hearts. 

However, clinical data on TA-NRP remain limited, with no multicenter, prospective, randomized controlled studies available. TA-NRP also faces significant ethical challenges [32]. Concerns arise from potential collateral blood flow providing cerebral perfusion despite clamping major aortic arch vessels, possibly leaving parts of the brain functional during TA-NRP perfusion. Although all DCD donors have a minimum 5-min interval of circulatory cessation before the second declaration of death, some brain regions may survive beyond this period [32]. This is why some U.S. medical institutions do not permit the use of TA-NRP.

## 6. Controlled Hypothermic Preservation Technology—SherpaPak Cardiac Transport System

Since the first heart transplant in 1967, traditional SCS has been the predominant method for preserving donor hearts for over 50 years [27]. SCS involves placing the donor heart in a sterile container with cold cardioplegic solution, surrounded by ice. Despite its simplicity and low cost, SCS has several drawbacks, including rapid and uneven cooling, unpredictable temperature fluctuations, and the risk of direct ice contact with myocardial tissue [33]. Temperatures below 2 °C can cause protein denaturation, cellular damage, irreversible diastolic dysfunction, and damage to the heart conduction system, potentially leading to graft failure [34]. Conversely, temperatures above 12 °C increase metabolic oxygen demand [35]. The International Society for Heart and Lung Transplantation (ISHLT) guidelines recommend maintaining donor heart temperature between 4 °C and 8 °C during transport [36], but this range is difficult to achieve consistently with traditional SCS. Additionally, an increasing number of donor hearts are recovered using TA-NRP technology, which involves warm ischemia and ischemia–reperfusion injury, making the myocardium more vulnerable and necessitating advanced preservation techniques.

**Figure 2:**
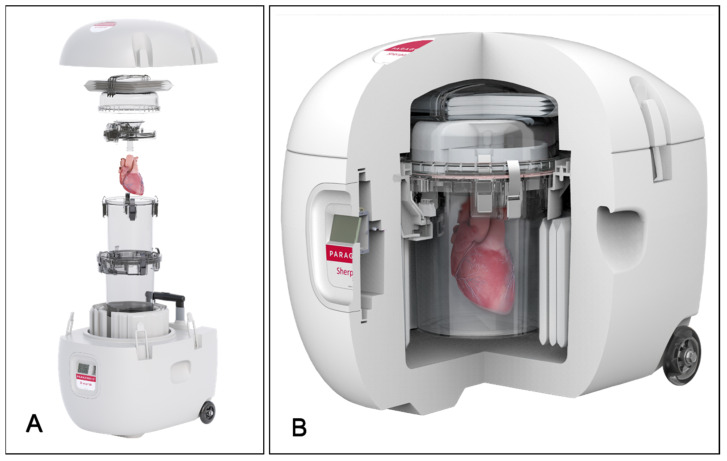
Paragonix SherpaPak Cardiac Transport System. (**A**). Exploded view of the SCTS, demonstrating its components and assembly process. (**B**). Cross-sectional view of the assembled SCTS, showing a preserved heart within the system. The SCTS employs controlled hypothermic preservation to maintain donor heart viability. The system ensures stable temperatures between 4 °C and 8 °C, minimizing myocardial damage during transport. The images are sourced from publicly available materials and are used with permission from Paragonix Technologies, Inc.

In 2018, the U.S. FDA approved a novel controlled hypothermic heart preservation system, the SherpaPak Cardiac Transport System (SCTS, Paragonix Technologies, Inc., Waltham, MA, USA). This commercial single-use device is designed to maintain a stable donor heart temperature between 4 °C and 8 °C for up to 40 h during transport [35]. The cooling mechanism employs special phase change materials that store and release large amounts of energy, providing uniform cooling and effectively preventing myocardial tissue damage due to freezing [35]. To use the system, the donor heart’s aorta is connected to the top of the inner canister via a connector, and the heart is fully immersed in the cardioplegic solution within the inner canister. The inner canister is then placed into the outer canister, which is equipped with cooling packs (phase change materials) and a protective outer shell (Figure 2). The device offers continuous real-time temperature monitoring of the donor heart, with data transmitted via Bluetooth to the user’s mobile phone. This information, including temperature and geographical location, can be shared in real time within the transplant medical team through an app.

In 2020, Dejan Radakovic and colleagues reported the first clinical study confirming the safety of using SCTS for donor heart preservation [37]. Subsequent studies have shown that SCTS reduces the incidence of PGD, decreases the need for pacemakers, and reduces postoperative red blood cell usage [38,39]. However, no randomized clinical trials have been conducted with SCTS. The GUARDIAN-Heart trial is an international multicenter registry comparing clinical outcomes of heart preservation using SCTS versus traditional SCS [40]. From 2015 to 2022, 255 recipients used SCTS, while 314 used SCS. The baseline characteristics of both groups were similar, though the distance between donor and recipient was longer in the SCTS group. Propensity score matching showed that SCTS significantly reduced severe PGD incidence (3.4% vs. 12.1%; *p* = 0.005) and improved 1-year survival rates (96.4% vs. 88.7%; *p* = 0.03). For ischemic times exceeding 4 h, SCTS reduced severe PGD (3.7% vs. 18.0%; *p* = 0.01) and improved 30-day survival rates (100% vs. 94.0%; *p* = 0.02). Although SCTS’s direct cost is higher than traditional SCS, GUARDIAN-Heart results suggest potential cost savings due to shorter ICU stays, fewer patients requiring MCS, and reduced severe PGD rates [41]. The latest data indicate that SCTS reduces severe PGD rates by 50% compared to traditional SCS (6.0% vs. 12.1%, *p* = 0.018) [33]. The longer the ischemic time, the higher the probability of severe PGD, but SCTS offers better outcomes across all ischemic times. Hearts preserved using SCS are more likely to develop severe PGD, with a 1-year mortality rate of 32.1% for recipients with severe PGD [33]. A study from Stanford University Hospital showed that prolonged ischemic time with traditional SCS is associated with increased recipient mortality, with an 11% increase in risk for every 10-min increase in ischemic time [39]. In contrast, SCTS use did not significantly impact mortality rates with prolonged ischemic time [39]. These studies suggest that SCTS offers benefits in reducing severe PGD and improving survival rates, particularly for longer ischemic times.

## 7. Hypothermic Oxygenated Perfusion (HOPE) Technology

**Figure 3:**
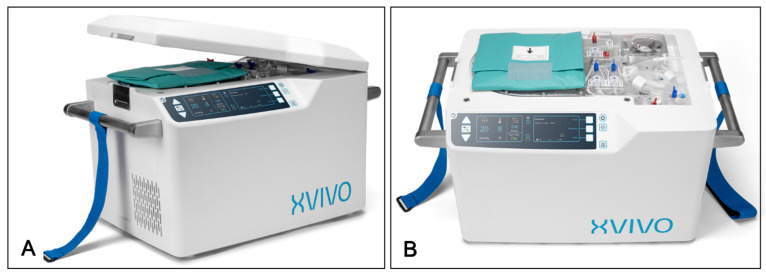
XVIVO Heart Perfusion System. (**A**). Side view with the cover. (**B**). Top view without the cover. The XVIVO Heart Perfusion System is designed for hypothermic oxygenated perfusion, providing continuous oxygen and nutrient supply to the donor heart. The images are used with permission from XVIVO Inc.

While the SCTS significantly improves heart preservation compared to traditional SCS, it remains a cold ischemic storage method. Cold ischemic storage has several negative effects on myocardial protection, including depletion of metabolic substrates, and endothelial cell damage, with cell swelling, edema, and acidosis being particularly prominent [42]. To address these issues and further enhance donor heart preservation, a clinical trial is currently underway in the United States using innovative HOPE technology, implemented via the XVIVO Heart Perfusion System (XVIVO Inc., Gothenburg, Sweden). The XVIVO system (Figure 3) is a portable device, functioning similarly to a miniature heart–lung machine. It continuously perfuses the donor heart with oxygen-rich, blood-containing cardioplegic solution at low temperatures, thereby reducing myocardial metabolic levels while providing continuous oxygenation and nutrient supply [43]. The system comprises a heart chamber, disposable perfusion unit, XVIVO perfusion solution, additives, and a gas tank. The heart chamber features automated pressure/flow control, gas exchange, leukocyte filter, cooling unit, battery, and software. The XVIVO perfusion solution, an albumin-based, hyperosmotic, nutrient-rich mixture, includes hormones and antibiotics and is combined with 300–500 mL of recipient-type red blood cells to achieve a hematocrit of 10–15%. After procurement, the ascending aorta is connected to the XVIVO-specific cannula and blood pump, with a left ventricular vent inserted through the mitral valve to prevent distension. The heart is placed in a reservoir, and coronary arteries are perfused antegradely through the ascending aorta cannula at 20 mmHg and 8 °C, achieving a coronary flow rate of 150–200 mL/min. During perfusion, the heart remains in a non-beating, quiescent state.

Preclinical studies have confirmed that the XVIVO system maintains myocardial contractility and endothelial function integrity during 24-h heart preservation [44]. A non-randomized Phase II clinical trial compared six donor hearts preserved using XVIVO perfusion with 25 hearts preserved using SCS [45]. The median preservation time was 223 min for the XVIVO group and 194 min for the SCS group. At six months, the XVIVO group had a 100% event-free survival rate (including PGD, need for ECMO support within 7 days, or acute rejection above 2R), compared to 72% in the SCS group. Another non-randomized, single-arm, multicenter study evaluated the impact of HOPE on extending preservation time (6 to 8 h) on 30-day recipient survival and post-transplant heart function [43]. The long-term preservation group had an average time of 414 min, with a maximum of 527 min, achieving a 100% 30-day recipient survival rate. Compared to the ISHLT registry control group, the extended preservation time did not adversely affect survival rates, which were significantly better than those of the control group. The study indicated that HOPE could extend heart preservation time to nearly 9 h, facilitating ultra-long-distance organ procurement. Compared to ex vivo NMP technology, HOPE offers potential advantages for long-term preservation of DBD donor hearts [43]. Longer preservation times with NMP technology increase the incidence of PGD, possibly due to myocardial edema, which is not observed with the XVIVO system. If the XVIVO perfusion system fails, preservation can switch from HOPE to SCS, making it safer during transport. Additionally, XVIVO does not require complex parameter adjustments during perfusion, making it simpler to use. Despite these advantages, HOPE cannot assess graft viability through lactate levels, hemodynamics, or heart contractility. The clinical trial for XVIVO is ongoing in the United States, but it has not yet seen widespread use.

## 8. Application of Extended-Criteria Donors, Post-Cardiopulmonary Resuscitation Donors, and High-Risk Donors

ECD hearts, also known as marginal donor hearts, include those with prolonged ischemic times, advanced age, a history of coronary artery disease (CAD), and reduced LVEF [46]. Historically, ECD hearts were linked to higher postoperative complications and mortality compared to standard donor hearts [47]. However, advancements in medical technology and the ongoing shortage of donor hearts have led to a reevaluation of ECD hearts, with an increasing number of transplants in the United States utilizing them. Current consensus suggests that ECD hearts can provide survival benefits for specific recipient populations. Research indicates that hearts from donors over 40 years of age are associated with poorer post-transplant survival outcomes, with the negative impact of donor age on recipient survival increasing with age [48]. The poorest outcomes are observed in recipients of hearts from donors aged 55 and older [48]. Nonetheless, critically ill recipients have shown survival benefits when using hearts from older donors [48]. A study from Stanford University Hospital found that recipients over 60 years old had similar average survival and transplant failure rates when using hearts from donors over 50 compared to younger donors [49]. Generally, older donor age is linked to poorer recipient survival, likely due to the increased risk of cardiac allograft vasculopathy, yet these donor hearts can still be suitable for specific candidates. Donors with CAD represent another category of ECDs. Studies suggest that hearts with mild to moderate CAD do not adversely affect recipient survival nor accelerate the development of allograft vasculopathy [50]. Jahanyar et al. analyzed the UNOS database from 1987 to 2017 and found no differences in median survival time, 5-year, or 10-year survival rates between recipients of hearts with (*n* = 650, 7.5%) and without (*n* = 7952, 92.5%) CAD, as confirmed by coronary angiography [51]. However, these studies lack detailed information on whether the donor required coronary artery bypass graft surgery [50,51], so the use of donor hearts with CAD is approached cautiously even in experienced transplant centers. Other re-evaluated ECDs include hearts with reduced LVEF and left ventricular hypertrophy (LVH). Studies show that reduced LVEF does not negatively impact recipient mortality post-transplant, with temporary declines in LV systolic function typically normalizing after transplantation [52,53]. Particularly for young, male DBD donors, even with reduced LVEF, the use of their hearts should be actively considered. Similarly, heart transplants using LVH donor hearts have shown comparable clinical outcomes to those using standard donor hearts [54]. Consequently, the use of LVH donor hearts is increasingly accepted as an effective way to expand the donor pool. However, caution is still necessary when LVH donors have additional comorbidities or high-risk factors, such as hypertension or advanced age.

In the United States, most donors undergo varying durations of cardiopulmonary resuscitation (CPR) before organ donation. Although CPR can reduce the acceptance rate of donor hearts, extensive research indicates that CPR does not affect the quality of the donor heart or the clinical outcomes for the recipient [55]. Analysis of UNOS data shows similar survival rates for heart transplant recipients from donors who underwent CPR and those who did not at 30 days (95.2% vs. 94.7%), 1 year (88.2% vs. 87.7%), and 5 years (72.8% vs. 74.2%) [56,57]. While recent studies suggest that CPR durations exceeding 55 min may correlate with poorer post-transplant survival rates [58], this conclusion remains debatable [59]. The current consensus is that a comprehensive echocardiographic evaluation of the heart is the most critical factor in deciding whether to accept a donor heart. CPR duration is a consideration but should not be decisive. ISHLT guidelines recommend that even if the donor CPR duration exceeds 30 min, with a satisfactory echocardiographic assessment of heart function and hemodynamics, this should not negatively impact recipient survival or outcomes [60]. The guidelines emphasize the importance of a thorough assessment of the donor heart post-CPR, rather than strictly adhering to CPR duration limits. Further research is needed to determine whether CPR duration alone can independently predict adverse outcomes in recipients.

High-risk organ donors carry a higher risk of transmitting infectious diseases to recipients. With advancements in treating human immunodeficiency virus and hepatitis C virus (HCV), the use of high-risk donor organs has increased. Studies show that recipients of high-risk donor hearts have similar 1-year (84.3% vs. 83%) and 5-year (71.2% vs. 65.5%) survival rates compared to those receiving standard-risk donor hearts [61,62]. For example, in 2006, recipients of HCV-positive donor hearts had more than double the 1-year, 5-year, and 10-year mortality rates compared to recipients of HCV-negative donor hearts [63], primarily due to liver disease and allograft vasculopathy. At that time, interferon and ribavirin were the only treatments for HCV. In 2014, new antiviral drug regimens were introduced, offering higher efficacy across multiple HCV genotypes, fewer side effects, and achieving sustained virological response in over 90–95% of HCV patients [64]. Consequently, more U.S. transplant centers began using organs from HCV-positive donors. In 2016, only seven heart transplant centers used HCV-positive donors, but by 2019, this number increased to 57 [65]. The proportion of HCV-positive heart donors rose from 0.6% in 2016 to 11.45% in 2019 [65], and the percentage of heart transplant candidates willing to accept HCV-positive donor hearts increased from less than 20% before 2016 to nearly 40% in 2018 [66]. According to UNOS data, the number of HCV-positive donor hearts used in 2021, 2022, and 2023 was 387, 420, and 458, respectively. The use of HCV-positive donors has shortened waiting times for recipients and increased the number of heart transplants without adversely affecting post-transplant mortality rates [67].

## 9. Future Directions

A breakthrough in the U.S. is the use of gene-edited pig hearts for human transplants. In January 2022, Dr. Griffith and his colleagues at the University of Maryland successfully performed the first heart transplant from a 10-gene-edited pig to a human [68], followed by a second transplant in September 2023. Although both recipients did not survive long-term due to complications such as viremia, antibody-mediated rejection, and ischemia–reperfusion injury [69,70], and despite being far from widespread clinical application, this technology represents a promising direction that could fundamentally address donor shortages.

Another significant research direction is how to evaluate donor heart quality and enhance myocardial protection ex vivo to reduce PGD incidence and severity. Despite extensive research, PGD remains a significant challenge, affecting both early mortality and long-term graft survival [71]. Factors such as donor age, preservation techniques, ischemic time, and pre-donation health conditions are strongly associated with PGD. Moreover, DCD and DBD hearts undergo different pathophysiological processes and preservation methods, leading to distinct etiological causes of PGD. DCD donors experience warm ischemia and hypoxemia, often using technologies like the TransMedics OSC or normothermic regional perfusion, while DBD donors experience a catecholamine surge due to brainstem death and typically use cold static preservation. Research shows that DCD recipients tend to have more severe biventricular PGD but shorter post-transplant MCS durations and hospital stays compared to DBD recipients, indicating differences in donor characteristics and recovery mechanisms [72]. To identify which donor hearts are more prone to PGD, discovering a combination of highly sensitive biomarkers is a promising direction. Future research should focus on biomarkers in the preservation solution or perfusate of donor hearts, using high-throughput omics technologies to identify markers for different causes and severities of PGD. For instance, several myocardial metabolites are valuable for grading PGD severity or distinguishing between PGD characteristics in DBD and DCD hearts [73]. If highly sensitive and specific biomarkers can be identified, they could be used not only for clarifying the mechanisms of PGD and evaluating the quality of donor hearts but also as targets for interventions to improve heart quality ex vivo. This would ultimately lead to significantly improved heart transplant outcomes.

Furthermore, the application of artificial intelligence (AI) in organ evaluation and procurement is another critical direction. Heart transplantation involves complex interactions among multiple factors. AI can process high-dimensional and intricate data, capturing patterns and relationships that traditional statistical methods might miss. Additionally, AI can dynamically learn and update with new data, continuously improving performance and accuracy. For example, an artificial neural network model can predict PGD risk by incorporating 33 significant predictors from 77 variables, emphasizing factors like donor age, cause of donor death, and ischemia time [74]. This underscores AI’s potential to optimize donor heart selection, identify high-risk factors, comprehensively evaluate heart quality, and predict post-transplant outcomes. However, current AI models face limitations due to missing data, data imbalances, and uneven variable distribution in datasets [75]. Establishing standardized multicenter databases that include comprehensive clinical and biosample data is urgently needed. Despite these challenges, advancing AI in this field is a crucial and promising direction for the future.

## 10. Conclusions

Over the past decade, significant advancements in donor heart procurement techniques have enabled highly individualized approaches. The primary goal remains the preservation and maintenance of energy stores and the integrity of both endothelium and myocardium, primarily through the careful avoidance of ischemic reperfusion damage. Among the emerging technologies, there is no definitive consensus on a superior method, as each presents unique advantages and disadvantages. The optimal approach depends on various factors, including donor type (DBD or DCD), transport distance, ischemic time, donor heart age and condition, surgical complexity, cost, and the procurement team’s experience. By considering these variables, clinicians can tailor their approach to each case, ensuring the best possible outcomes. This personalized strategy underscores the importance of flexibility and adaptability in heart transplantation, enhancing success rates and expanding the donor pool to meet patient needs. Looking ahead, leveraging AI to determine the optimal recovery method based on donor heart characteristics and automatically control recovery systems like OCS, along with employing biological technologies to evaluate and improve donor heart quality ex vivo, are promising possibilities. These potential advancements will significantly increase the quantity and quality of donor hearts, ultimately enhancing overall heart transplant outcomes. 

## Data Availability

Not applicable.

## References

[1] Martin S.S., Aday A.W., Almarzooq Z.I., Anderson C.A., Arora P., Avery C.L., Baker-Smith C.M., Barone Gibbs B., Beaton A.Z., Boehme A.K. (2024). 2024 Heart Disease and Stroke Statistics: A Report of US and Global Data from the American Heart Associati on. Circulation.

[2] Barnard C.N. (1967). The operation. A human cardiac transplant: An interim report of a successful operation performed at Groote Schuur Hospital, Cape Town. S. Afr. Med. J..

[3] Zhu Y., Lingala B., Baiocchi M., Arana V.T., Williams K.M., Shudo Y., Oyer P.E., Woo Y.J. (2021). The Stanford experience of heart transplantation over five decades. Eur. Heart J..

[4] Colvin M.M., Smith J.M., Ahn Y.S., Handarova D.K., Martinez A.C., Lindblad K.A., Israni A.K., Snyder J.J. (2024). OPTN/SRTR 2022 Annual Data Report: Heart. Am. J. Transplant..

[5] Jaiswal A., Gadela N.V., Baran D., Balakumaran K., Scatola A., Radojevic J., Gluck J., Arora S., Hammond J., Ali A. (2021). Clinical outcomes of older adults listed for heart transplantation in the United States. J. Am. Geriatr. Soc..

[6] Wever-Pinzon O., Edwards L.B., Taylor D.O., Kfoury A.G., Drakos S.G., Selzman C.H., Fang J.C., Lund L.H., Stehlik J. (2017). Association of recipient age and causes of heart transplant mortality: Implications for personalization of post-transplant management-An analysis of the International Society for Heart and Lung Transplantation Registry. J. Heart Lung Transplant..

[7] Awad M.A., Shah A., Griffith B.P. (2022). Current status and outcomes in heart transplantation: A narrative review. Rev. Cardiovasc. Med..

[8] Schroder J.N., Scheuer S., Catarino P., Caplan A., Silvestry S.C., Jeevanandam V., Large S., Shah A., MacDonald P., Slaughter M.S. (2023). The American Association for Thoracic Surgery 2023 Expert Consensus Document: Adult cardiac transplantation utilizing donors after circulatory death. J. Thorac. Cardiovasc. Surg..

[9] Chew H.C., Iyer A., Connellan M., Scheuer S., Villanueva J., Gao L., Hicks M., Harkness M., Soto C., Dinale A. (2019). Outcomes of Donation after Circulatory Death Heart Transplantation in Australia. J. Am. Coll. Cardiol..

[10] Schroder J.N., Patel C.B., DeVore A.D., Bryner B.S., Casalinova S., Shah A., Smith J.W., Fiedler A.G., Daneshmand M., Silvestry S. (2023). Transplantation Outcomes with Donor Hearts after Circulatory Death. N. Engl. J. Med..

[11] Chen Q., Emerson D., Megna D., Osho A., Roach A., Chan J., Rowe G., Gill G., Esmailian F., Chikwe J. (2023). Heart transplantation using donation after circulatory death in the United States. J. Thorac. Cardiovasc. Surg..

[12] Urban M., Moody M., Lyden E., Kinen L., Castleberry A.W., Siddique A., Lowes B.D., Stoller D.A., Lungren S.W., Um J.Y. (2023). Impact of donation after circulatory death heart transplantation on waitlist outcomes and transplantation activity. Clin. Transplant..

[13] Smith D.E., Kon Z.N., Carillo J.A., Chen S., Gidea C.G., Piper G.L., Reyentovich A., Montgomery R.A., Galloway A.C., Moazami N. (2022). Early experience with donation after circulatory death heart transplantation using normothermic regional perfusion in the United States. J. Thorac. Cardiovasc. Surg..

[14] Madan S., Saeed O., Forest S.J., Goldstein D.J., Jorde U.P., Patel S.R. (2022). Feasibility and Potential Impact of Heart Transplantation From Adult Donors after Circulatory Death. J. Am. Coll. Cardiol..

[15] Gernhofer Y.K., Bui Q.M., Powell J.J., Perez P.M., Jones J., Batchinsky A.I., Glenn I.C., Adler E., Kearns M.J., Pretorius V. (2023). Heart transplantation from donation after circulatory death: Impact on waitlist time and transplant rate. Am. J. Transplant..

[16] Hornby L., Dhanani S., Shemie S.D. (2018). Update of a Systematic Review of Autoresuscitation after Cardiac Arrest. Crit. Care Med..

[17] Croome K.P., Barbas A.S., Whitson B., Zarrinpar A., Taner T., Lo D., MacConmara M., Kim J., Kennealey P.T., Bromberg J.S. (2023). American Society of Transplant Surgeons recommendations on best practices in donation after circulatory death organ procurement. Am. J. Transplant..

[18] Joshi Y., Villanueva J., Gao L., Hwang B., Zhao C., Doyle A., Wu J., Jansz P., Macdonald P. (2022). Donation after Circulatory Death: A New Frontier. Curr. Cardiol. Rep..

[19] Shudo Y., Benjamin-Addy R., Koyano T.K., Hiesinger W., MacArthur J.W., Woo Y.J. (2021). Donors after circulatory death heart trial. Future Cardiol..

[20] Lerman J.B., Agarwal R., Patel C.B., Keenan J.E., Casalinova S., Milano C.A., Schroder J.N., DeVore A.D. (2024). Donor Heart Recovery and Preservation Modalities in 2024. JACC Heart Fail..

[21] White C.W., Ambrose E., Müller A., Li Y., Le H., Hiebert B., Arora R., Lee T.W., Dixon I., Tian G. (2015). Assessment of donor heart viability during ex vivo heart perfusion. Can. J. Physiol. Pharmacol..

[22] Fedson S. (2024). Heart transplant donation after circulatory death: Current status and implications. Curr. Opin. Cardiol..

[23] Langmuur S.J.J., Amesz J.H., Veen K.M., Bogers A., Manintveld O.C., Taverne Y. (2022). Normothermic Ex Situ Heart Perfusion with the Organ Care System for Cardiac Transplantation: A Meta-analysis. Transplantation.

[24] Schroder J.N., Patel C.B., DeVore A.D., Casalinova S., Koomalsingh K.J., Shah A.S., Anyanwu A.C., D’Alessandro D.A., Mudy K., Sun B. (2024). Increasing Utilization of Extended Criteria Donor Hearts for Transplantation: The OCS Heart EXPAND Trial. JACC Heart Fail..

[25] Kothari P. (2023). Ex-Vivo Preservation of Heart Allografts-An Overview of the Current State. J. Cardiovasc. Dev. Dis..

[26] Urban M., Ryan T.R., Um J.Y., Siddique A., Castleberry A.W., Lowes B.D. (2024). Financial impact of donation after circulatory death heart transplantation: A single-center analysis. Clin. Transplant..

[27] Kounatidis D., Brozou V., Anagnostopoulos D., Pantos C., Lourbopoulos A., Mourouzis I. (2023). Donor Heart Preservation: Current Knowledge and the New Era of Machine Perfusion. Int. J. Mol. Sci..

[28] Joyce D.L., Carlson S.F., Kohmoto T., Durham L., Ubert A., Candek C., Koerten D., Joyce L.D. (2022). Thoracoabdominal Normothermic Regional Perfusion for Cardiac Procurement. ASAIO J..

[29] Alamouti-Fard E., Garg P., Wadiwala I.J., Yazji J.H., Alomari M., Hussain W.A., Elawady M.S., Jacob S. (2022). Normothermic Regional Perfusion is an Emerging Cost-Effective Alternative in Donation after Circulatory Death (DCD) in Heart Transplantation. Cureus.

[30] Hoffman J.R., McMaster W.G., Rali A.S., Rahaman Z., Balsara K., Absi T., Levack M., Brinkley M., Menachem J., Punnoose L. (2021). Early US experience with cardiac donation after circulatory death (DCD) using normothermic regional perfusion. J. Heart Lung Transplant..

[31] Messer S., Cernic S., Page A., Berman M., Kaul P., Colah S., Ali J., Pavlushkov E., Baxter J., Quigley R. (2020). A 5-year single-center early experience of heart transplantation from donation after circulatory-determined death donors. J. Heart Lung Transplant..

[32] Entwistle J.W., Drake D.H., Fenton K.N., Smith M.A., Sade R.M., Cardiothoracic Ethics F. (2022). Normothermic Regional Perfusion: Ethical Issues in Thoracic Organ Donation. Ann. Thorac. Surg..

[33] D’alessandro D., Schroder J., Meyer D.M., Vidic A., Shudo Y., Silvestry S., Leacche M., Sciortino C.M., Rodrigo M.E., Pham S.M. (2024). Impact of controlled hypothermic preservation on outcomes following heart transplantation. J. Heart Lung Transplant..

[34] Hendry P.J., Walley V.M., Koshal A., Masters R.G., Keon W.J. (1989). Are temperatures attained by donor hearts during transport too cold?. J. Thorac. Cardiovasc. Surg..

[35] Michel S.G., LaMuraglia Ii G.M., Madariaga M.L., Anderson L.M. (2015). Innovative cold storage of donor organs using the Paragonix Sherpa Pak devices. Heart Lung Vessel..

[36] Copeland H., Hayanga J.A., Neyrinck A., MacDonald P., Dellgren G., Bertolotti A., Khuu T., Burrows F., Copeland J.G., Gooch D. (2020). Donor heart and lung procurement: A consensus statement. J. Heart Lung Transplant..

[37] Radakovic D., Karimli S., Penov K., Schade I., Hamouda K., Bening C., Leyh R.G., Aleksic I. (2020). First clinical experience with the novel cold storage SherpaPak system for donor heart transportation. J. Thorac. Dis..

[38] Bitargil M., Haddad O., Pham S.M., Garg N., Jacob S., Ahmed M.M.E., Landolfo K., Patel P.C., Goswami R.M., Moreno J.C.L. (2022). Packing the donor heart: Is SherpaPak cold preservation technique safer compared to ice cold storage. Clin. Transplant..

[39] Zhu Y., Shudo Y., He H., Kim J.Y.P.-C., Elde S., Williams K.M., Walsh S.K.B., Koyano T.K.B., Guenthart B., Woo Y.J. (2023). Outcomes of Heart Transplantation Using a Temperature-controlled Hypothermic Storage System. Transplantation.

[40] Shudo Y., Leacche M., Copeland H., Silvestry S., Pham S.M., Molina E., Schroder J.N., Sciortino C.M., Jacobs J.P., Kawabori M. (2023). A Paradigm Shift in Heart Preservation: Improved Post-transplant Outcomes in Recipients of Donor Hearts Preserved with the SherpaPak System. ASAIO J..

[41] Voigt J.D., Leacche M., Copeland H., Wolfe S.B., Pham S.M., Shudo Y., Molina E., Jacobs J.P., Stukov Y., Meyer D. (2023). Multicenter Registry Using Propensity Score Analysis to Compare a Novel Transport/Preservation System to Traditional Means on Postoperative Hospital Outcomes and Costs for Heart Transplant Patients. ASAIO J..

[42] Belzer F.O., Southard J.H. (1988). Principles of solid-organ preservation by cold storage. Transplantation.

[43] McGiffin D.C., Kure C.E., Macdonald P.S., Jansz P.C., Emmanuel S., Marasco S.F., Doi A., Merry C., Larbalestier R., Shah A. (2024). Hypothermic oxygenated perfusion (HOPE) safely and effectively extends acceptable donor heart preservation times: Results of the Australian and New Zealand trial. J. Heart Lung Transplant..

[44] Steen S., Paskevicius A., Liao Q., Sjoberg T. (2016). Safe orthotopic transplantation of hearts harvested 24 hours after brain death and preserved for 24 hours. Scand. Cardiovasc. J..

[45] Nilsson J., Jernryd V., Qin G., Paskevicius A., Metzsch C., Sjöberg T., Steen S. (2020). A nonrandomized open-label phase 2 trial of nonischemic heart preservation for human heart transplantation. Nat. Commun..

[46] Critsinelis A.C., Patel S., Nordan T., Chen F.Y., Couper G.S., Kawabori M. (2023). Trends in Outcomes of Heart Transplants Using Extended Criteria Donors: A United Network for Organ Sharing Database Analysis. Ann. Thorac. Surg..

[47] Felker G.M., Milano C.A., Yager J.E., Hernandez A.F., Blue L., Higginbotham M.B., Lodge A.J., Russell S.D. (2005). Outcomes with an alternate list strategy for heart transplantation. J. Heart Lung Transplant..

[48] Weber D.J., Wang I.-W., Gracon A.S.A., Hellman Y.M., Hormuth D.A., Wozniak T.C., Hashmi Z.A. (2014). Impact of donor age on survival after heart transplantation: An analysis of the United Network for Organ Sharing (UNOS) registry. J. Card. Surg..

[49] Shudo Y., Guenther S.P., Lingala B., He H., Hiesinger W., MacArthur J.W., Currie M.E., Lee A.M., Boyd J.H., Woo Y.J. (2020). Relation of Length of Survival after Orthotopic Heart Transplantation to Age of the Donor. Am. J. Cardiol..

[50] Lechiancole A., Vendramin I., Sponga S., Sappa R., Zanuttini D., Spedicato L., Ferrara V., Di Nora C., Livi U. (2021). Influence of donor-transmitted coronary artery disease on long-term outcomes after heart transplantation—A retrospective study. Transpl. Int..

[51] Jahanyar J., Liao J.M., Zhang N., Butterfield R.J., Hardaway B.W., Scott R.L., Steidley E.D. (2019). Does Pre-Existing Donor Heart Coronary Artery Disease Impact Survival after Orthotopic Heart Transplantation?. J. Heart Lung Transplant..

[52] Sibona A., Khush K.K., Oyoyo U.E., Martens T.P., Hasaniya N.W., Razzouk A.J., Bailey L.L., Rabkin D.G. (2019). Long-term transplant outcomes of donor hearts with left ventricular dysfunction. J. Thorac. Cardiovasc. Surg..

[53] Chen C.W., Sprys M.H., Gaffey A.C., Chung J.J., Margulies K.B., Acker M.A., Atluri P. (2017). Low ejection fraction in donor hearts is not directly associated with increased recipient mortality. J. Heart Lung Transplant..

[54] Pinzon O.W., Stoddard G., Drakos S.G., Gilbert E.M., Nativi J.N., Budge D., Bader F., Alharethi R., Reid B., Selzman C.H. (2011). Impact of donor left ventricular hypertrophy on survival after heart transplant. Am. J. Transplant..

[55] McCulloch M.A., Zuckerman W.A., Möller T., Knecht K., Lin K.Y., Beasley G.S., Peng D.M., Albert D.C., Miera O., Dipchand A.I. (2020). Effects of donor cause of death, ischemia time, inotrope exposure, troponin values, cardiopulmonary resuscitation, electrocardiographic and echocardiographic data on recipient outcomes: A review of the literature. Pediatr. Transplant..

[56] Quader M.A., Wolfe L.G., Kasirajan V. (2013). Heart transplantation outcomes from cardiac arrest-resuscitated donors. J. Heart Lung Transplant..

[57] Galeone A., Varnous S., Lebreton G., Barreda E., Hariri S., Pavie A., Leprince P. (2017). Impact of cardiac arrest resuscitated donors on heart transplant recipients’ outcome. J. Thorac. Cardiovasc. Surg..

[58] Kulshrestha K., Greenberg J.W., Guzman-Gomez A.M., Kennedy J.T., Hossain M., Zhang Y., Zafar F., Morales D.L. (2024). Up to an Hour of Donor Resuscitation Does Not Affect Pediatric Heart Transplantation Survival. Ann. Thorac. Surg..

[59] Leon M., Shudo Y. (2024). Optimizing Donor Heart Utilization Amidst Organ Shortage: Feasibility of Using Hearts Post-Long CPR. Ann. Thorac. Surg..

[60] Copeland H., Knezevic I., Baran D.A., Rao V., Pham M., Gustafsson F., Pinney S., Lima B., Masetti M., Ciarka A. (2023). Donor heart selection: Evidence-based guidelines for providers. J. Heart Lung Transplant..

[61] Gaffey A.C., Doll S.L., Thomasson A.M., Venkataraman C., Chen C.W., Goldberg L.R., Blumberg E.A., Acker M.A., Stone F., Atluri P. (2016). Transplantation of “high-risk” donor hearts: Implications for infection. J. Thorac. Cardiovasc. Surg..

[62] Gaffey A.C., Cucchiara A.J., Goldberg L.R., Blumberg E.A., Acker M.A., Atluri P. (2016). Transplantation of Center for Disease Control “High-Risk” Donor Hearts Does Not Adversely Impact Long-Term Outcomes in Adults. J. Card. Fail..

[63] Gasink L.B., Blumberg E.A., Localio A.R., Desai S.S., Israni A.K., Lautenbach E. (2006). Hepatitis C virus seropositivity in organ donors and survival in heart transplant recipients. JAMA.

[64] Aslam S., Grossi P., Schlendorf K.H., Holm A.M., Woolley A.E., Blumberg E., Mehra M.R. (2020). Utilization of hepatitis C virus-infected organ donors in cardiothoracic transplantation: An ISHLT expert consensus statement. J. Heart Lung Transplant..

[65] Huckaby L.V., Seese L.M., Handzel R., Wang Y., Hickey G., Kilic A. (2021). Center-level Utilization of Hepatitis C Virus-positive Donors for Orthotopic Heart Transplantation. Transplantation.

[66] Wang J., Gustafson S.K., Skeans M.A., Lake J.R., Kim W.R., Kasiske B.L., Israni A.K., Hart A. (2020). OPTN/SRTR 2018 Annual Data Report: Hepatitis C. Am. J. Transplant..

[67] Gernhofer Y.K., Brambatti M., Greenberg B.H., Adler E., Aslam S., Pretorius V. (2019). The impact of using hepatitis c virus nucleic acid test-positive donor hearts on heart transplant waitlist time and transplant rate. J. Heart Lung Transplant..

[68] Mohiuddin M.M., Singh A.K., Scobie L., Goerlich C.E., Grazioli A., Saharia K., Crossan C., Burke A., Drachenberg C., Oguz C. (2023). Graft dysfunction in compassionate use of genetically engineered pig-to-human cardiac xenotransplantation: A case report. Lancet.

[69] Peterson L., Yacoub M.H., Ayares D., Yamada K., Eisenson D., Griffith B.P., Mohiuddin M.M., Eyestone W., Venter J.C., Smolenski R.T. (2024). Physiological basis for xenotransplantation from genetically modified pigs to humans. Physiol. Rev..

[70] Schmauch E., Piening B., Mohebnasab M., Xia B., Zhu C., Stern J., Zhang W., Dowdell A.K., Kim J.I., Andrijevic D. (2024). Integrative multi-omics profiling in human decedents receiving pig heart xenografts. Nat. Med..

[71] Brahmbhatt D.H., Blitzer D., Billia F., Copeland H. (2023). Acute complication posttransplant: Primary allograft dysfunction. Curr. Opin. Organ. Transplant..

[72] Ayer A., Truby L.K., Schroder J.N., Casalinova S., Green C.L., Bishawi M.A., Bryner B.S., Milano C.A., Patel C.B., Devore A.D. (2023). Improved Outcomes in Severe Primary Graft Dysfunction after Heart Transplantation Following Donation after Circulatory Death Compared with Donation after Brain Death. J. Card. Fail..

[73] Truby L.K., Kwee L.C., Bowles D.E., Casalinova S., Ilkayeva O., Muehlbauer M.J., Huebner J.L., Holley C.L., DeVore A.D., Patel C.B. (2024). Metabolomic profiling during ex situ normothermic perfusion before heart transplantation defines patterns of substrate utilization and correlates with markers of allograft injury. J. Heart Lung Transplant..

[74] Linse B., Ohlsson M., Stehlik J., Lund L.H., Andersson B., Nilsson J. (2023). A machine learning model for prediction of 30-day primary graft failure after heart transplantation. Heliyon.

[75] Grzyb C., Du D., Nair N. (2024). Artificial Intelligence Approaches for Predicting the Risks of Durable Mechanical Circulatory Support Therapy and Cardiac Transplantation. J. Clin. Med..

